# Polyphenols and Maillard Reaction Products in Dried *Prunus spinosa* Fruits: Quality Aspects and Contribution to Anti-Inflammatory and Antioxidant Activity in Human Immune Cells Ex Vivo

**DOI:** 10.3390/molecules27103302

**Published:** 2022-05-20

**Authors:** Anna Magiera, Monika Ewa Czerwińska, Aleksandra Owczarek, Anna Marchelak, Sebastian Granica, Monika Anna Olszewska

**Affiliations:** 1 Department of Pharmacognosy, Faculty of Pharmacy, Medical University of Lodz, 1 Muszynskiego St., 90-151 Lodz, Poland; aleksandra.owczarek@umed.lodz.pl (A.O.); anna.marchelak@umed.lodz.pl (A.M.); monika.olszewska@umed.lodz.pl (M.A.O.); 2 Department of Biochemistry and Pharmacogenomics, Medical University of Warsaw, 1 Banacha St., 02-097 Warsaw, Poland; monika.czerwinska@wum.edu.pl; 3 Centre for Preclinical Research, Medical University of Warsaw, 1B Banacha St., 02-097 Warsaw, Poland; 4 Microbiota Lab, Centre for Preclinical Studies, Department of Pharmacognosy and Molecular Basis of Phytotherapy, Medical University of Warsaw, 1 Banacha St., 02-097 Warsaw, Poland; sgranica@wum.edu.pl

**Keywords:** *Prunus spinosa* fruits, LC–MS, NMR, phenolics, 5-hydroxymethylfurfural, drying, ROS, inflammatory mediators

## Abstract

Dried *Prunus spinosa* fruits (sloes) are folk phytotherapeutics applied to treat chronic inflammatory disorders. However, their pharmacological potential, activity vectors, and drying-related changes in bioactive components remain unexplored. Therefore, the present research aimed to evaluate the anti-inflammatory and antioxidant effects of dried sloes in ex vivo models of human neutrophils and peripheral blood mononuclear cells (PMBCs) and establish their main active components. It was revealed that the fruit extracts significantly and dose-dependently inhibited the respiratory burst, downregulated the production of elastase (ELA-2) and TNF-α, and upregulated the IL-10 secretion by immune cells under pro-inflammatory and pro-oxidant stimulation. The slightly reduced IL-6 and IL-8 secretion was also observed. The structural identification of active compounds, including 45 phenolics and three Maillard reaction products (MRPs) which were formed during drying, was performed by an integrated approach combining LC-MS/MS, preparative HPLC isolation, and NMR studies. The cellular tests of four isolated model compounds (chlorogenic acid, quercetin, procyanidin B2, and 5-hydroxymethylfurfural), supported by statistical correlation studies, revealed a significant polyphenolic contribution and a slight impact of MRPs on the extracts’ effects. Moreover, a substantial synergy was observed for phenolic acids, flavonoids, condensed proanthocyanidins, and MPRs. These results might support the phytotherapeutic use of dried *P. spinosa* fruits to relieve inflammation and establish the quality control procedure for the extracts prepared thereof.

## 1. Introduction

*Prunus spinosa* L. (blackthorn) is a small to medium-sized thorny tree of the rose family, native to Europe, Western Asia, and Northern Africa, and locally naturalised in New Zealand, Tasmania, and eastern North America [1]. The plant is one of few wild rosaceous species yielding edible fruits. They are tiny, globose, purple plums, which, when fresh, are widely used to produce various preserves, such as jams, juices, wines, brandy or liquors, while when dried, they are added to herbal teas [2]. Moreover, blackthorn fruits (sloes), both fresh and dried, are traditional herbal medicines recommended mostly to treat chronic inflammation-related disorders, especially those within the gastrointestinal and urinary tracts, but also diarrhoea, hypertension, and metabolic diseases, such as diabetes and obesity [3,4,5,6].

Although ethnopharmacological sources do not differentiate the application range of fresh and dried sloes, to date scientific interest has focused almost exclusively on fresh fruits [7,8,9,10,11,12,13,14], and information on the dried *P. spinosa* plums is extremely scarce [15,16,17]. On the other hand, fresh sloes have been relatively deeply studied, primarily in terms of the mechanisms of their anti-inflammatory and antioxidant effects. In our previous study, we have documented that the fresh fruit extracts significantly modulate the functions of human immune cells, neutrophils and peripheral blood mononuclear cells (PBMCs) involved in the pathogenesis of inflammation. They downregulate the secretion of reactive oxygen species (ROS), pro-inflammatory cytokines (tumour necrosis factor α, TNF-α) and enzymes (elastase, ELA-2), and stimulate the production of the anti-inflammatory cytokine IL-10 [13]. Moreover, according to Sabatini et al. [14], the alcoholic extracts of fresh sloe plums can decrease the expression of IL-6 and adhesion molecules ICAM-1 and VCAM-1 in human endothelial cells (HUVEC). Furthermore, the high antioxidant potential of fresh *P. spinosa* plums was revealed in human malignant promonocytes [7] and non-cellular in vitro models [8,10,11,12].

The accumulated research suggests that the main active components of fresh sloes are polyphenols. A rich fraction of various polyphenols with more than 50 individual compounds has been revealed in the fresh plant material, including condensed proanthocyanidins, phenolic acids, anthocyanins, and flavonols [13]. It is known that the biological activity of fruit polyphenolic fractions depends on the presence of specific individual compounds and their proportions [18]. Post-harvest technological processing may influence these factors, leading to structural changes of polyphenols and changes in their quantity and bioavailability. For instance, thermal treatment can result in transformation/isomerisation, enzymatic breakdown, or degradation of labile compounds. Additionally, the release of polyphenols bound with polysaccharides, proteins, or lipids may occur during drying. All these changes may have a dualistic effect on the plant material’s final activity. On the one hand, the loss of some polyphenolic components may reduce its pharmacological potential, but on the other hand, the newly formed or released compounds may reveal higher biological effects [19,20,21,22].

Thermal fruit processing may also induce the formation of non-polyphenolic compounds, such as Maillard reaction products (MRPs). They are aromatic derivatives formed in a multistep, non-enzymatic reaction between amino acids and reducing sugars when dehydration occurs at high temperatures. Since MPRs from food, especially low-molecular-weight ones such as furans, are orally ingested by humans and absorbed into the bloodstream, their biological activity is of great interest [23]. To date, both the harmful properties (genotoxic, mutagenic, carcinogenic) and the beneficial health effects (antioxidant, anti-inflammatory, antibacterial) of these compounds have been revealed [24,25]. However, the reported data are conflicting, and further research is required to assess their pharmacological activity [23]. Moreover, the quantitative determination of MPRs in various processed plant materials is required to ensure their safety.

Extraction of a fruit matrix is crucial in developing fruit-based medicines and functional products [26]. The fractionated extraction enables the concentration of the active plant components and facilitates their identification through selective isolation and comparative activity studies [13,18].

Therefore, the aim of the present study was: (a) to characterise for the first time the phytochemical composition of dried *P. spinosa* fruits in the function of fractionated extraction with particular emphasis on individual polyphenols and MPRs and their drying-related molecular transformation; (b) to investigate the antioxidant and anti-inflammatory activity of the fruit extracts and their main pure components in human neutrophils and PBMCs ex vivo; (c) to select extracts with the highest potential as anti-inflammatory agents; and (d) to evaluate the contribution of polyphenols and MPRs to the observed biological effects. The phytochemical profiling was performed using various analytical techniques, including LC–MS/MS, preparative HPLC isolation, and subsequent spectroscopic identification (NMR) of primary MPRs formed during drying. The cellular tests covered the release assessment of a series of pro-oxidant, pro-inflammatory, and anti-inflammatory factors (ROS, TNF-*α*, ELA-2, IL-8, IL-6, and IL-10). Moreover, the cellular safety of the extracts and pure compounds was evaluated by flow cytometry.

## 2. Results and Discussion

### 2.1. Fractionated Extraction and Phytochemical Composition of the Extract/Fractions

As *P. spinosa* dried fruits are primarily used as a traditional herbal remedy [3,4,5], for the present study, the fresh fruits were dried in the typical conditions of herbal medicinal products [27]. The same fresh plant material batch and the same extraction procedure as in our previous work on fresh fruits [13] were used for direct result comparison. In the first step, the dried fruits were extracted with methanol-water (75:25, *v*/*v*) to maximise recovery of polyphenols [13] and reflect the composition of tinctures, the most popular medicinal preparations of dried sloes [3,4,5]. Then, the obtained source hydroalcoholic extract (MED) was partitioned by fractionated extraction to yield the fractions of diethyl ether (DEFD), ethyl acetate (EAFD), *n*-butanol (BFD), and water residue (WRD).

**Figure 1:**
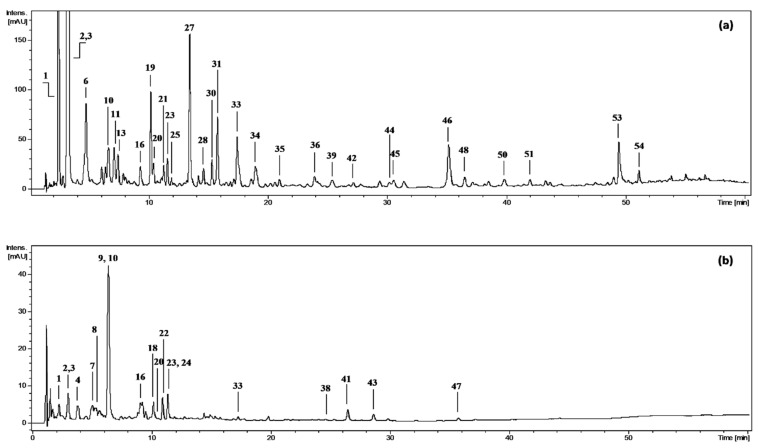
Representative UHPLC chromatograms at 280 nm of (**a**) diethyl ether fraction from dried fruits of *Prunus spinosa*, DEFD, and (**b**) *n*-butanol fraction from dried fruits, BFD. Peak numbers refer to those implemented in Table S1 (Supplementary Materials).

The qualitative LC-PDA-ESI-MS^3^ assay revealed the presence of 54 constituents in the extracts/fractions (Figure 1, peaks 1−54; Table S1, Figure S1), among which 45 polyphenols and four non-phenolic compounds were fully or tentatively identified by comparing their retention properties, UV–vis spectra, and MS/MS fragmentation patterns with reference compounds or literature data (Table S1).

#### 2.1.1. Polyphenolic Profile

The polyphenolic constituents represented two main classes: phenolic acids and aldehydes (27 analytes, peaks 4−6, 8−11, 14−17, 19−21, 23, 25−34, 36, 39) and flavonols (16 analytes, peaks 37, 38, 40−53). Moreover, one anthocyanin (24) and one flavanol (13) were found. Phenolic acids were the most structurally diversified group that comprised caffeoyl-, coumaroyl-, and ferulolylquinic acids (8, 10, 16, 20, 21, 23, 26, 32); caffeoylquinic acid hexoside (9); shikimic acid derivatives (14, 30, 33, 34, 36, 39); *p*-coumaric acid (31) and its hexoside (17); caffeic acid hexoside (25); and benzoic acid derivatives including *p*-hydroxybenzoic acid (11), protocatechuic acid (6) and its hexoside (5), vanillic acid (19) and its hexoside (4) and malate hexoside (15). The phenolic aldehyde (27) was identified as vanillin. Among flavonols, one aglycone quercetin (53), thirteen quercetin mono-, di-, and triglycosides (38, 40−48), including malyl (49, 50) and acetyl esters (52), one kaempferol hexoside (51) and one dihydrokaempferol hexoside (aromadendrin hexoside, 37) were found. The sole anthocyanin detected (24) was cyanidin 3-*O*-*β*-D-glucopyranoside, while the single flavanol (13) was tentatively identified as an (epi)catechin derivative.

The polyphenolic profiles of the extracts/fractions from dried sloes differed significantly from those observed previously for fresh fruits [13]. With 45 versus 57 identified compounds, the dried sloes revealed a less rich composition and lesser variety of flavonols (17 vs. 24 analytes) and anthocyanins (one vs. four compounds). Moreover, the dried material was distinguished by the loss of 19 constituents present in fresh fruits and the appearance of seven polyphenols (13, 14, 17, 29, 37, 39, 51) newly formed during drying and thus detected for the first time in *P. spinosa*. The lost compounds, including several derivatives of sinapic and caffeic acids, as well as isorhamnetin, kaempferol, cyanidin, and peonidin glycosides, occurred in the fresh material at low or trace levels and were probably destroyed by thermal processing. Some other compounds decomposed partly; for example, cleavage of glycosidic bonds might explain the appearance of kaempferol hexoside (51) instead of two kaempferol di- and triglycosides from fresh fruits. Moreover, partial isomerisation of pseudodepsides could also take place; an example is the case of *p*-coumaroylshikimic acids (36, 39), among which one was present in both materials (36) but second only in dried sloes (39). Furthermore, three other drying-related processes, such as the oxidative coupling of caffeoylquinic acids [28], depolymerisation of condensed proanthocyanidins [21], and recovery of polyphenols from lignified stone tissue [29] were probably responsible for the presence of caffeoylquinic acid dehydrodimer (29), (epi)catechin derivative (13), and aromadendrin hexoside (37) in dried fruits, respectively.

The quantitative profiles of polyphenols in the extracts/fractions of dried fruits differed significantly depending on the extraction solvent (Table 1). The total phenolic levels amounted to 26.8−124.0 mg GAE/g dw (TPC, determined by the Folin-Ciocalteu method) and 9.6−109.1 mg/g dw (TPH, assayed by HPLC–PDA), with the highest values observed for DEFD and EAFD. In all extracts/fractions (except EAFD), the TPC levels exceeded the TPH values, determined as the sum of individual low-molecular-weight polyphenols, including total phenolic acids (TPA), total flavonoids (TFL), and total anthocyanins (TAC). For instance, the TPC value in the source extract MED was about 3-times higher than TPH. This gap might be explained by the presence of high-molecular-weight tannin-type proanthocyanidins, which are undetectable by standard RP-LC procedures due to high polarity. Therefore, their content was estimated by a procyanidin-specific spectrophotometric assay (TTC). Consequently, the sum TPH+TTC better represents the genuine total phenolic contents of the sloe extracts. A similar relationship was previously revealed for the fresh fruit extracts [13], which means that drying did not affect the TPC/TPH ratio. Similarly, as in the fresh fruit [13], only one low-molecular-weight flavan-3-ol was detected, which is also in accordance with the works of Guimarães et al. [9] and Mikulić-Petkovsek et al. [12], who did not detect such compounds in sloes at all or only a few representatives.

**Figure 2:**
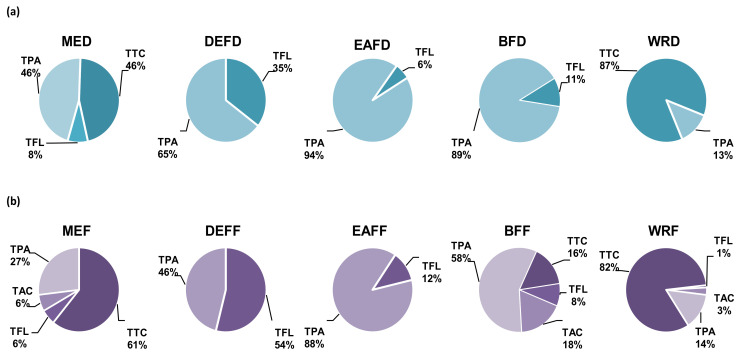
Contribution of individual groups of compounds to total phenolic contents (TPH+TTC) in *Prunus spinosa* fruit extracts: (**a**) dried fruits and (**b**) fresh fruits. TPA, TAC, and TFL: total contents of phenolic acids, anthocyanins, and flavonoids, respectively, determined by HPLC-PDA; TTC: total content of condensed tannins in procyanidin B2 (PB2) equivalents; TPH, sum of individual polyphenols by HPLC. For extract codes, see Table 1 and Section Abbreviations. The extracts/fractions from dried fruits were coded by the last letter “D”, while those from fresh fruits by “F”. For detailed experimental data on fresh fruits, see Magiera et al. [13].

On the other hand, the thermal treatment significantly influenced the levels and proportions between individual compounds and their fractions (Figure 2). The major compounds occurring in MED were phenolic acids and tannins, while flavonoids constituted less than 10% of the extract, and anthocyanins were below the detection limits (Table 1, Figure 2). Partitioning of MED between solvents of different polarities led to the concentration of phenolic acids in DEFD, EAFD, and BFD, tannins in WRD, and flavonoids mainly in DEFD. Anthocyanins were found in trace amounts only in BFD. The TPA levels varied between 0.8−102.5 mg/g dw with the peak values in EAFD, where derivatives of phenolic acids prevailed (constituted 93% TPH+TTC). The most abundant compounds from this group were pseudodepsides of quinic acids, especially chlorogenic acids (10, 20, 23), forming 37−100% TPAs. Among the individual analytes, cryptochlorogenic acid (23) dominated in EAFD with a level of 50.8 mg/g dw, while in the other extracts/fractions, the primary compound was neochlorogenic acid (10) with the peak content in BFD (16.2 mg/g dw). Apart from pseudodepsides, the fruits also contained simple benzoic and cinnamic acids. Only traces of these compounds were found in the source extract, but they were concentrated in DEFD, where protocatechuic acid (6) and vanillic acid (22) formed 40% TPAs. The flavonoid contents amounted to 0.3−41.1 mg/g dw, with the highest values in DEFD, where TFL formed 32% TPH+TTC. The particular extracts/fractions significantly varied in the flavonoid profiles. Quercetin (53) dominated in DEFD (formed 63% TFL), whereas glycosides prevailed in other fractions: isoquercitrin (quercetin 3-*O*-*β*-D-glucopyranoside, 42) in EAFD (16% TFL), rutin (quercetin 3-*O*-(6′′-*O*-*α*-L-rhamnopyranosyl)-*β*-D-glucopyranoside, 41) in BFD (68% TFL), and avicularin (quercetin 3-*O*-*α*-L-arabinofuranoside, 46) in MED (45% TFL). Regarding tannins, they are only present in MED and WRD but at relatively high levels of 46% and 87% TTC+TPH, respectively.

The qualitative and quantitative profiles of bioactive fruit components may change during thermal processing [30]. The present research showed for the first time that sloes dried in the conditions typical for the production of herbal medicines contain lower levels of all types of polyphenols compared to fresh fruits [13]. Among different compounds, anthocyanins and proanthocyanidins changed most dramatically, and their total levels in the dried fruits dropped by almost 100% and 76%, respectively (Table S2, Supplementary Materials). This observation is consistent with the earlier reports on the relatively low thermal stability of anthocyanins and proanthocyanidins in various fruits, including grapes, bilberries, blueberries, and domesticated plums [19,31,32]. Phenolic acids and flavonols are less sensitive to drying [33], which also seems true in sloes, as their total amounts decreased by 46% and 56%, respectively (Table S2). Interestingly, in contrast to the majority of most individual polyphenols, the levels of some simple phenolic acids (e.g., protocatechuic, vanillic, *p*-coumaric, and *p*-hydroxybenzoic acids) and cryptochlorogenic acid were higher in dried than in fresh fruits. The first case suggests that cleavage of some phenolic esters might have taken place during drying. In the second case, as the levels of other chlorogenic acid isomers decreased in line with the general trend, their partial isomerisation might be one possible explanation for the higher content of cryptochlorogenic acid in the dried fruits. All these changes corresponded to the differences in the TPC and TPH levels, which in dried fruits were about 3-times lower than in fresh sloes (Table S2). Nevertheless, the TPC levels of dried blackthorn fruits (0.55 g GAE/100 g fw and 1.3 g GAE/100 g dw) were similar to or surpassed the values reported for fruits valued in phytotherapy for their beneficial health effects and anti-inflammatory activity, such as cranberries (0.45 g/100 g fw), blueberries (0.13 g/100 g fw) or hawthorn fruits (2.1 g GAE/100 g dw) [12,15].

#### 2.1.2. Non-Phenolic Compounds

**Figure 3:**
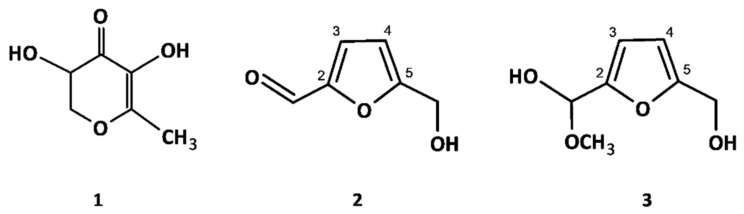
Structures of MRPs identified in dried *Prunus spinosa* fruits.

Apart from polyphenols, the dried blackthorn fruits were also distinguished by the presence of traces of a cyanogenic glycoside amygdalin (18) and three MPRs formed by dehydration of sugars and Maillard reaction, including 3-hydroxy-2,3-dihydromaltol (1) and two furfural derivatives (peak 2 and 3). The last two compounds had to be isolated for structural identification. The isolation was carried out by preparative HPLC. The isolates were obtained with high yield and identified after thorough spectral profiling (^1^H NMR, ^13^C NMR, COSY, HMBC, and HMQC). Their structures and NMR data were shown in Figure 3 and Table 2.

The ^1^H NMR spectra of compound 3 revealed the presence of two one-proton doublets at *δ* 6.31 ppm and *δ* 6.39 ppm with a small coupling constant (*J* = 3.0 Hz). The heteronuclear correlation experiments (HSQC, HMBC) showed one-bond correlations of the proton resonances with the carbon signals at *δ* 108.8 ppm and *δ* 107.6 ppm, respectively, and two-bond correlations with carbon resonances at *δ* 155.2 ppm and *δ* 154.2 ppm, respectively. This set of signals is characteristic of a 2,5-disubstituted furan ring [34]. The substituent at C-2 was interpreted as a –CH_2_OH group due to a two-proton singlet at *δ* 4.52 ppm in the ^1^H NMR spectrum and a corresponding ^13^C NMR carbon resonance at *δ* 67.0 ppm. The substituent at C-5 gave in ^1^H NMR one-proton singlet at *δ* 5.41 ppm with the corresponding carbon signal at *δ* 98.2 ppm, suggesting the presence of a –CH(OH)_2_ group. Additionally, the proton resonance at *δ* 3.37 ppm (3H, *s*) correlated with the carbon signal of the –CH(OH)_2_ group, which indicated that one of the hydroxyl groups is methylated. Eventually, compound 3 was identified as (5-hydroxymethylfur-2-yl)-methoxymethanol [35]. The spectral data of compound 2 were similar to those of compound 3. However, all the ^1^H NMR resonances of the furan ring were downshifted, suggesting at least one substituent of higher electronegativity. Indeed, the ^1^H NMR high-field signal of the substituent in the C-5 position at *δ* 9.56 ppm (1H, *s*) and its corresponding ^13^C carbon resonance at *δ* 182.6 ppm indicated an aldehyde group. Thus, compound 2 was identified as 5-hydroxymethylfurfural (HMF) [36].

All three MRPs were revealed for the first time in *P. spinosa* fruits but are known in numerous domesticated *Prunus* species [33]. The MRPs have also been found in various food products like other dried fruits, juices, coffee, bakery products, or honey. These by-products, particularly HMF, have gained much interest due to the doubt on their safety and health potential [37]. The toxicological effects of HMF, such as hepato-, nephro-, cy-to-, and genotoxic effects, as well as mutagenic and carcinogenic properties, have been preliminarily demonstrated in experimental animals and cultured mammalian cells [37,38,39], however, the reported data are inconclusive [23]. Because of the potential high exposure and possible toxicity of MRPs, their content in processed foods and medicinal products, should be monitored to assess health hazards. Specific, accurate methods should thus be developed and validated for the task. In the present study, the HPLC-PDA method, previously developed for the quality control of fresh sloes [13], was revalidated for the dried fruit matrix, and MRPs were determined in all extracts/fractions thereof (Table 1), with the peak concentration in DEFD and EAFD. The content of these compounds in sloes was 460 mg/kg dw (Table S2), which is within the wide range of 1−2200 mg/kg dw reported for other commonly consumed dried fruits generally recognised as safe, such as apples, apricots, dates, and plums [25,33,40]. Moreover, blackthorn fruits contained significantly lower MPRs levels than typically observed (1100−2200 mg/kg dw) for widely consumed prunes (from *Prunus domestica*) [40]. However, further studies on the biological effects of MRPs are required to verify their safety.

### 2.2. Bioactivity of the Extracts/Fractions and Model Compounds in Human Immune Cells Ex Vivo

#### 2.2.1. Cellular Models

Accumulated research strongly suggests that low-grade inflammation is a critical player in the progression of chronic human disorders, such as bowel diseases, vascular abnormalities, cancer, diabetes, obesity, metabolic syndrome, and neurodegenerative diseases [41]. In humans, chronic inflammatory processes are linked with continuous up-regulation of pro-oxidant and pro-inflammatory functions of immune cells, primarily neutrophils and PBMCs differentiating into macrophages. Upon activation, the immune cells produce and release a wide range of pro-inflammatory and pro-oxidant mediators, including chemokines/cytokines (i.e., IL-8, IL-6, TNF-α), tissue-remodelling enzymes (i.e., ELA-2) and ROS, which collectively contribute to tissue dysfunction [42]. On the other hand, these cells can also secrete some anti-inflammatory factors (such as IL-10), which help to limit inflammation [43]. Considering their primary and complementary roles in inflammatory reactions, both neutrophils and PBMCs were chosen as cellular models for the present ex vivo study of antioxidant and anti-inflammatory effects of *P. spinosa* fruit extracts. Because sloes are recommended by traditional medicine to treat various types of inflammatory disorders, both local in the digestive tract and systemic [3,4,5,6], the extracts were tested in a wide concentration range of 1−100 µg/mL to cover the polyphenolic levels potentially achievable in the bloodstream (1−5 µg/mL) and gastrointestinal tract (25−100 µg/mL) after oral intake of polyphenol-rich products. A broader discussion on the bioavailability of orally administrated polyphenols was published earlier [13,44]. Apart from the extracts, pure chlorogenic acid, quercetin, procyanidin B2, and HMF were tested as model compounds, representative of the fruit phenolic acids, flavonoids, proanthocyanidins, and MPRs.

#### 2.2.2. Effects on Viability of Neutrophils and PBMCs

The potential cytotoxicity of the model compounds and extracts/fractions was checked by flow cytometry and propidium iodide staining. The viability of neutrophils and PBMCs after 24−48 h of incubation with the analytes, except for DEFD at 50−100 µg/mL, was 85.0−95.1% and did not differ significantly (*p* > 0.05) from that of LPS-stimulated (91.8−96.4%) and control, non-stimulated (92.9−94.4%) cells (Figure S2). Only for DEFD at the highest concentration, the cells’ viability was slightly lower and amounted to 76.9−83.5%. Nevertheless, all analytes might be regarded as non-cytotoxic to the tested cells.

#### 2.2.3. Influence on Pro-Oxidant Functions of Human Neutrophils: Antioxidant Effect

**Figure 4:**
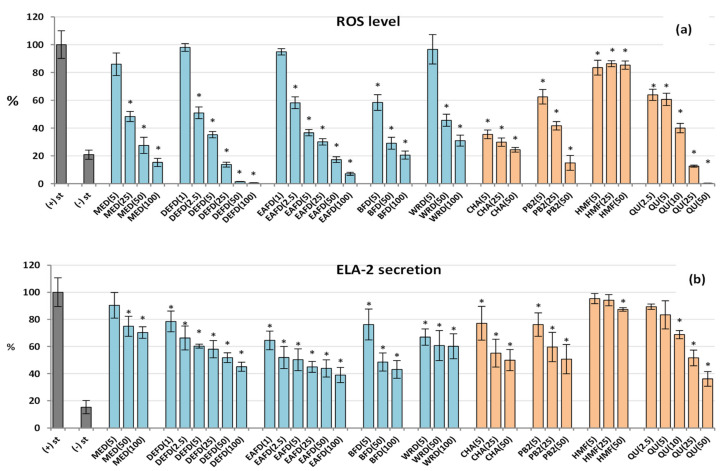
Effect of fruit extracts/fractions (1–100 µg/mL) and standards (2.5–50 µM) on the release of (**a**) ROS, reactive oxygen species; (**b**) ELA-2, neutrophils elastase from human neutrophils. Pure compounds: CHA, chlorogenic acid; PB2, procyanidin B2; HMF, 5-hydroxymethylfurfural; QU, quercetin (positive control). For extracts codes see Table 1. Data expressed as means ± SD of five independent experiments performed with cells isolated from five independent donors. Statistical significance in Dunnett’s test: * *p* < 0.05 compared with the stimulated control.

Neutrophils are the most abundant immune cells in human blood and one of the first responders to pro-inflammatory stimuli. The activated neutrophils start the process of respiratory burst that results in excessive production of ROS, especially O_2_^•−^, which is then transformed into other highly toxic molecules, including ^•^OH, H_2_O_2_, NO^•^, ONOO^−^, and HClO. These oxidants form an aggressive cocktail that may generate persistent oxidative stress, leading to chronic inflammation [42]. The elevated levels of ROS and oxidative stress-related biomarkers are observed in the blood or inflamed tissues of patients with various chronic diseases, including digestive tract inflammatory disorders [45], diabetes and its vascular complications [46], which are within the traditional application range of sloes [3,4,5,6]. The accumulated research on fresh *P. spinosa* fruits strongly suggests that their antioxidant activity at the cellular level might be partly responsible for their beneficial health effects [7,13,14]. The present study monitored the impact of the dried sloe extracts on an oxidative burst of the neutrophils using luminol as a universal detector of the whole array of ROS produced in this process. The results revealed for the first time that dried sloes also exhibit significant and concentration-dependent antioxidant effects in cellular models. All analysed extracts/fractions reduced the ROS production in *f*-MLP-stimulated human neutrophils by up to 69−99% at 100 μg/mL (Figure 4a). Moreover, the source extract MED (the extractable dried fruit matrix) effectively inhibited the ROS release at 25–100 μg/mL (*p* < 0.05), limiting at the highest concentration the ROS levels to their physiological values observed for non-stimulated control neutrophils. Out of the tested extracts, the DEFD and EAFD (the polyphenol-richest fractions) were the most active, interfering with the ROS secretion by at least 99% at 100 μg/mL and 49% at 2.5 μg/mL. It might suggest the substantial contribution of polyphenols to the observed antioxidant effects. Indeed, the linear correlation between the ROS release and total phenolic levels, both TPC and TPH+TTC, was negative and statistically significant (*r*_max_ = −0.7099, *p* < 0.001; Table S3). In contrast, the correlations for the polyphenolic subgroups (TTC, TPA, TFL) were insignificant (*p* > 0.05), but the potent antioxidant effects of the model polyphenols (chlorogenic acid, procyanidin B2, and quercetin) at even the lowest tested concentration of 2.5–5 µM (0.8–2.9 μg/mL) might indicate the synergic contribution of all relevant classes of polyphenols to the cellular antioxidant activity of the extracts, with the most outstanding impact of phenolic acids and tannins due to their prevalence in the extracts (Figure 2). For instance, the most active chlorogenic acid lowered the ROS release to 65% at 5 µM and 21% at 50 µM (Figure 4a).

#### 2.2.4. Effect on the ELA-2 Release by Human Neutrophils

After stimulation, neutrophils release a wide range of tissue-remodelling enzymes potentiating inflammation by increasing the extracellular matrix permeability for pro-inflammatory mediators. As a key enzyme hydrolysing elastin, one of the major extracellular matrix components, ELA-2 triggers numerous inflammatory diseases, e.g., it impairs regeneration of the colon mucosa in digestive tract disorders [47] and contributes to the pathological vascular permeability in diabetes [48]. As shown in Figure 4b, the dried fruit extracts significantly and in a dose-dependent manner inhibited the ELA-2 generation by the *f*MLP+cytochalasin B-stimulated neutrophils. The most potent inhibitory effect was detected for the polyphenolic-richest fractions DEFD, EAFD, and BFD, active even at low concentrations of 1−5 µg/mL (*p* < 0.05). For instance, EAFD downregulated the ELA-2 secretion by 35% at 1 µg/mL and 50% at 5 µg/mL. Similarly, to the case of ROS production, the strong activity of chlorogenic acid, procyanidin B2, and quercetin at 5–10 µM (3.0–5.8 µg/mL) and the significant correlation between the phenolic levels (TPC or TPH+TTC) and ELA-2 release (*r*_max_ = −0.6510, *p* < 0.005; Table S3) suggested the synergic contribution of phenolic acids, tannins, and flavonoids to the observed effects. The most active polyphenol in the test was quercetin, which reduced the ELA-2 secretion to 64% at 50 µM compared to the stimulated control.

#### 2.2.5. Influence on the Cytokine Secretion by Human Neutrophils and PBMCs

**Figure 5:**
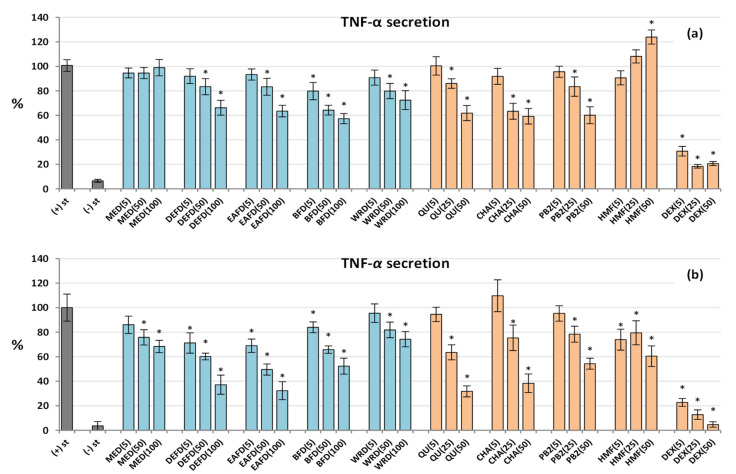
Effect of fruit extracts/fractions (1–100 µg/mL) and standards (5–50 µM) on the release of tumour necrosis factor α (TNF-α) from stimulated human immune cells: (**a**) neutrophils; and (**b**) PBMCs. Standards: QU, quercetin; CHA, chlorogenic acid; PB2, procyanidin B2; HMF, 5-Hydroxymethylfurfural. Positive control: DEX, dexamethasone. For extracts codes see Table 1. Data expressed as means ± SD of five independent experiments performed with cells isolated from five independent donors. Statistical significance in Dunnett’s test: * *p* < 0.05 compared with the stimulated control.

Cytokines are small signalling proteins and primary controllers of the growth and activity of immune cells. Several different cell types coordinate their functions as a part of the immune system, including neutrophils and PBMCs differentiating into macrophages [49]. The master cytokine in the network is TNF-α – a priming agonist of immune cells and a pro-inflammatory mediator orchestrating the neutrophil-PBMC/macrophage interplay during inflammation [50]. As shown in Figure 5, all analysed extracts/fractions (except MED in neutrophils) downregulated the TNF-α secretion in both cell types at 50–100 µg/mL (*p* < 0.05) with a more robust response from LPS-stimulated PBMCs, the leading TNF-α producers in humans. For instance, DEFD at 100 µg/mL reduced the TNF-α release by 34% in neutrophils and 63% in PBMCs. Interestingly, some variations in the anti-TNF-α activity of individual extracts in both cellular models existed: the most active were DEFD and EAFD in PBMCs but BFD in neutrophils. Moreover, in PBMCs, a significant reduction in the TNF-α level (*p* < 0.05) was also seen for DEFD, EAFD, and BFD at 5 µg/mL, while in neutrophils, only BFD revealed a similar effect. The extracts’ effects reflected the activity of pure compounds, which were also more effective in PBMCs than in neutrophils. Interestingly, all three pure polyphenols revealed comparable effects in neutrophils, while quercetin and chlorogenic acid were slightly more potent than procyanidin B2 in PBMCs. Nevertheless, the synergistic effects of all polyphenols might be concluded from the significant correlations between the activity parameters, TPC and TPH+TTC values of the extracts/fractions (*r*_min_ = −0.9116 in PBMCs, *p* < 0.001; Table S3).

Apart from TNF-α, the impact of the extracts/fractions on the secretion of two other pro-inflammatory (IL-8, IL-6) and one anti-inflammatory interleukin (IL-10) was tested. IL-8, also known as the neutrophil chemotactic factor, stimulates the migration and adhesion of neutrophils to the endothelium [42]. IL-6, released mostly by monocytes and macrophages, affects various immune system functions, including the production of neutrophils in the bone marrow. Monocytes/macrophages also release IL-10, the most important endogenous inhibitor of cytokine synthesis that ensures protection from the over-exuberant inflammatory response and associated tissue damage [51].

**Figure 6:**
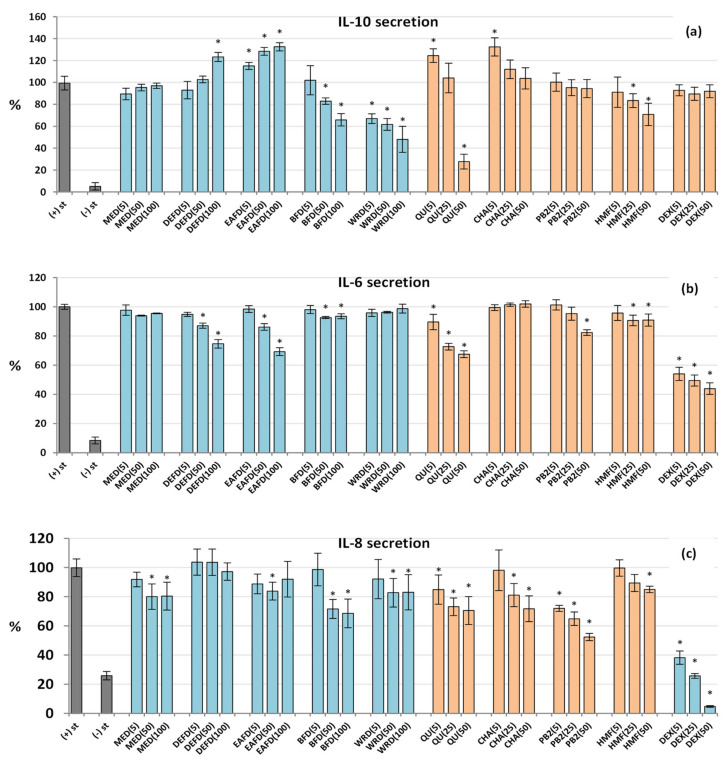
Effects of fruit extracts/fractions (5–100 µg/mL) and standards (5–50 µM) on the secretion of anti-inflammatory and pro-inflammatory interleukins from the stimulated human immune cells: (**a**) IL-10 from PBMCs; (**b**) IL-6 from PBMCs; and (**c**) IL-8 from neutrophils. Standards: QU, quercetin; CHA, chlorogenic acid; PB2, procyanidin B2; HMF, 5-Hydroxymethylfurfural. Positive control: DEX, dexamethasone. For extracts codes see Table 1. Data expressed as means ± SD of five independent experiments performed with cells isolated from five independent donors. Statistical significance in Dunnett’s test: * *p* < 0.05 compared with the stimulated control.

As shown in Figure 6, the analysed extracts/fractions affected mainly the IL-10 secretion, while their effects on IL-8 and IL-6 were less pronounced. Interestingly, the extracts modulated the IL-10 release in different directions: DEFD and EAFD up-regulated its secretion, BFF and WRF inhibited its production, while MED did not alter this cytokine release compared to the LPS-stimulated cells (Figure 6a). The stimulatory effect reached a maximum (33% increase) for EAFD, while the inhibitory effect (52% decrease) was maximal for WRD at 100 µg/mL. Moreover, EAFD and WRF revealed significant effects at 5−50 µg/mL (*p* < 0.05).

Together with the activity of model polyphenols, this dichotomy suggested that only low-molecular-weight polyphenols, especially phenolic acids and flavonoids (abundant in DEFD and EAFD, and represented by chlorogenic acid and quercetin at lower levels), might contribute to the potentiated IL-10 secretion. Consequently, a significant correlation between the phenolic contents and IL-10 secretion (*r*_min_ = 0.5549, *p* < 0.05) was primarily due to the substantial stimulatory effects of DEFD and EAFD, relatively the richest in phenolic acids and flavonoids (Table 2, Figure 2). It might reinforce the suggestion of their primary contribution to the up-regulation of IL-10. In contrast, condensed procyanidins (represented by procyanidin B2) or some non-phenolic compounds (enriched in hydrophilic fractions, especially WRF) might act as IL-10 inhibitors. The structure and activity profile of these compounds should be explained in detail in future studies.

Concerning IL-8 and IL-6, the extracts/fractions tended to inhibit their secretion from both LPS-stimulated neutrophils and PBMCs (Figure 6b,c), but the statistically significant effects were visible (*p* < 0.05) only for selected fractions or at elevated concentration levels of 50–100 µg/mL. BFD downregulated the IL-8 release most strongly among the extracts, by 31% at 100 µg/mL, while DEFD and EAFD were the most influential anti-IL-6 factors, inhibiting its production by 25% and 31%, respectively. The correlation tests confirmed that the production of IL-6 was inversely associated with the TPC and TPH+TTC values (*r*_min_ = –0.9378, *p* < 0.001; Table S3). Surprisingly, pure polyphenols, especially procyanidin B2, exhibited relatively potent activity in the IL-8 secretion test, which resulted in a lack of correlation between phenolic levels and activity parameters (Table S3). It might suggest some interference from non-phenolic compounds, which requires future studies.

Dexamethasone (DEX), a potent anti-inflammatory drug (5−50 µM), significantly (*p* < 0.05) reduced the release of all pro-inflammatory cytokines in both cellular models but did not influence (*p* > 0.05) the IL-10 production in PBMCs (Figure 5 and Figure 6).

#### 2.2.6. Drying-Related Differences in Biological Capacity of Dried and Fresh Fruits

A comparison of the results obtained for dried sloes with those presented earlier for fresh blackthorn fruits [13] indicated that drying-related changes in their polyphenolic composition significantly influenced their anti-inflammatory and antioxidant activity. In general, the dried fruit extracts presented slightly lower activity, and the degradation of anthocyanins and partial loss of proanthocyanidins seem to be of key importance for their reduced effectiveness. For instance, in the ROS and TNF-α secretion tests in neutrophils, the extracts/fractions in which the TAC and TTC contents fell the most (MED, BFD, WRD) were up to 19% and 21% less active, respectively, compared to the respective fractions of fresh fruits (MEF, BFF, WRF). On the other hand, the partial flavonoid degradation was probably responsible for the lowered effects of anthocyanin- and procyanidin-free fractions (DEFD/EAFD vs. DEFF/EAFF). For example, a 37% slide of TFL in DEFD vs. DEFF resulted in an 18% lower stimulation of IL-10 production in PBMCs. However, despite variations in their phytochemical profiles, the activity differences between dried and fresh fruit extracts are not critical, and the dried fruits retained the ability to significantly modulate the inflammatory response of both types on immune cells.

#### 2.2.7. Biological Effects of HMF

The present study is the first attempt to assess the effects of HMF, a model MRP, on the viability and pro-inflammatory and pro-oxidant functions of human neutrophils and PBMCs. As shown in Figure S2, the viability of HMF-treated cells was 85.1–86.6% and 94.9–95.5% for neutrophils and PBMCs, respectively, and did not differ significantly from the control range. Therefore, HMF at 5−50 µM (0.6–6.0 µg/mL) can be considered non-cytotoxic to the tested immune cells. As the average exposure to HMF in a regular human diet is 44 µM per day [52], this observation might be useful for the general assessment of HMF-related health hazards. It is compatible with the recent reports [53] pointing to significant antiproliferative effects of HMF on human gastric mucosal epithelial cells only in concentrations higher than 4 mM (504 µg/mL).

Figure 4a showed that HMF at 5−50 µM decreased the ROS generation by LPS-stimulated neutrophils by about 15%. At the highest tested concentration (50 µM), HMF also slightly (by 9−15%) reduced the secretion of some pro-inflammatory factors, including ELA-2, IL-6, and IL-8 (Figure 4b and Figure 6b,c). On the other hand, the compound significantly downregulated the release of the anti-inflammatory cytokine IL-10 by up to 29% (Figure 6a). Concerning TNF-α secretion, the HMF effects varied with the cell type (Figure 5); the secretion of this cytokine increased in neutrophils (by up to 24% at 50 µM) but decreased in PBMCs (by up to 40% at 50 µM), which may be due to differences in the cytokine production mechanisms in various cells. In comparison to model polyphenols, HMF was distinguished by the stimulation of the TNF-α production in neutrophils and anti-IL-10 effects in PBMCs. Because the extract richest in HMF and MRPs (DEFD) revealed the opposite trend, the contribution of these compounds to the biological effects of dried sloes in terms of IL-10 secretion might be considered negligible or reversed by the presence of other constituents. On the other hand, HMF might be co-responsible for the downregulation of TNF-α release by PBMCs.

The observed low antioxidant potential of HMF is consistent with the previous reports of Kong et al. [54], which demonstrated that this compound (0.25−1 µM) reduced the ROS production in the murine macrophage-like cell line RAW 264.7 by about 10%. However, in the same cellular model, HMF at 1 µM strongly inhibited the production of IL-1β, IL-6, and TNF-α, even by more than 50% in the case of IL-6 [54]. Moreover, Uchida et al. [55] showed that HMF could downregulate the IL-6 secretion from bone-marrow-derived murine mast cells by up to 70% at high concentrations (200 µg/mL, 1.59 mM). Interestingly, our results revealed only a 10% decrease in the IL-6 level in human PBMCs treated with 50 µM HMF, which strongly supports the use of human cells in biological activity studies of foods and herbal medicines.

## 3. Materials and Methods

### 3.1. Plant Material and Extracts Preparation

Mature fruits of *P. spinosa* were collected in October 2018 by handpicking from bushes naturally growing in Krasnobród (50° 32′ 41″N and 23° 12′ 55″E), Poland (near the Roztocze National Park). Prof. M. A. Olszewska authenticated the fruit sample, according to Popescu and Caudullo [1]. The voucher specimens (no. KFG/HB/18006-PSP-FRUIT) were deposited in the Herbarium of the Department of Pharmacognosy, Medical University of Lodz (Poland). After harvesting, the raw material was divided into two parts. One of them (fresh fruits) was processed as described earlier [13], and the second was dried in conditions typical for the production of herbal medicinal products (convective drying at a temperature not higher than 60°C) [27]. The dried material was powdered with an electric grinder and sieved through a 0.750-mm sieve. A portion (500.1 g) was sequentially refluxed with methanol-water (75:25, *v*/*v*; 3 × 1.5 L, 30 min, and 2 × 20 min) to give the hydroalcoholic extract (MED, 250.9 g dw). Next, the sample of MED (220.0 g dw) was suspended in warm (40 °C) water (1.5 L) and partitioned with organic solvents (10 × 200 mL each) to yield diethyl ether fraction (DEFD, 7.8 g dw), ethyl acetate fraction (EAFD, 2.7 g dw), *n*-butanol fraction (BFD, 23.7 g dw) and water residue (WRD, 26.3 g dw).

### 3.2. Qualitative and Quantitative Analysis of Phenolic Compounds

The profile of individual phenolic compounds in the extracts/fractions was evaluated by UHPLC-PDA-ESI-MS^3^ and HPLC-PDA using the same analytical procedures and equipment applied previously to fresh fruits [13]. Calibration for HPLC-PDA quantitative analysis was performed using 21 authentic standards, according to Marchelak et al. [56] and Magiera et al. [13]. The validation parameters for 5-hydroxymethylfurfural (HMF) – dried fruit-specific standard (not involved in the previous studies) were shown in Table 3. The identified peaks were quantified as equivalents of external standards per dw of the extracts, depending on their structures and UV-Vis spectra. Briefly, hydroxybenzoic acid derivatives were quantified as protocatechuic acid; monocaffeoylquinic acid isomers as chlorogenic acid; dicaffeoylquinic acid isomers as cynarin; other hydroxycinnamic acid derivatives as caffeic acid or *p*-coumaric acid; flavonoid mono- and diglycosides as isoquercitrin and rutin, respectively; anthocyanins as cyanidin 3-*O*-glucopyranoside; and MRPs as HMF. Results were calculated per dry weight (dw) of the extracts.

The total phenolic content (TPC) and total content of tannin-type proanthocyanidins (TTC) were quantified by the Folin-Ciocalteu method and vanillin assay, respectively, as described previously [13]. The results were expressed in mg gallic acid (GAE)/g of extract dw and mg procyanidin B2 (PB2)/g of extract dw, respectively.

The quantitative results were recalculated for the fresh weight (fw) of fruits using the extraction yield of MED. All standards used for phytochemical profiling were of HPLC grade (purity at least 95%) and were purchased from Sigma-Aldrich (St. Louis, MO, USA) or previously isolated in our laboratory from *P. spinosa* flowers and leaves [13 and references therein].

### 3.3. Isolation and Structure Elucidation of MRPs

For isolation purposes, the preparative sample of dried blackthorn fruits (600.5 g) was extracted as described in Section 3.1 to give the methanol-water (75:25, *v*/*v*) extract (295.7 g dw). The crude extract was suspended in warm (40 °C) water (1.8 L) and re-extracted with ethyl acetate (10 × 250 mL each) to yield the ethyl acetate fraction (5.6 g dw). Two MRPs (compounds 2 and 3; Figure 2) were isolated by preparative HPLC-PDA using a CombiFlash EZ Prep chromatograph (Teledyne ISCO, Lincoln, NE, USA) equipped with a PDA detector, a self-priming pump, and an internal fraction collector. Separations were performed on a C18 RediSep Prep column (5.0 μm, 150 mm × 20 mm; Teledyne ISCO, Lincoln, NE, USA). The ethyl acetate fraction (5.0 g dw) was dissolved in 40 mL DMSO, filtered through a polytetrafluoroethylene (PTFE) syringe filter (25 mm, 0.2 µm, Ahlstrom, Helsinki, Finland), and the filtrate was injected directly in 2 mL portions into the HPLC system using the manual injection.

The elution system consisted of solvent A (water) and solvent B (acetonitrile) with the elution profile as follows: 0–1 min, 5% B (*v*/*v*); 1–5 min, 5–10% B; 5–10 min, 10% B; 15–15 min, 10−100% B; 15–20 min, 100% B; 20 min, 5% B; 20–28 min, 5% B (equilibration). The flow rate was 19.0 mL/min, and the column was maintained at 25 °C. The effluent was monitored at 290 nm (MRPs) and 350 nm (flavonoids) and automatically collected into 20 mL test tubes. The subfractions enriched in MRPs were combined and lyophilised to yield a mixture of compounds 2 and 3 (450.0 mg) in a brown, amorphous, semi-solid substance.

The structures of the isolates were elucidated by 1D and 2D NMR experiments. The NMR spectra (^1^H NMR, ^13^C NMR, COSY, HMBC, and HMQC) were acquired on a Bruker Avance III 600 MHz NMR spectrometer (Bruker BioSpin, Rheinstetten, Germany) in methanol-d4 (600 MHz for ^1^H and 150.9 MHz for ^13^C) using TMS as the internal standard. All reagents were from Sigma-Aldrich (St. Louis, MO, USA).

### 3.4. Biological Activity Studies in Cellular Models

#### 3.4.1. Isolation of Neutrophils and PBMCs from Human Buffy Coats

Neutrophils and PBMCs were isolated from human buffy coats by dextran sedimentation, erythrocyte lysis, and centrifugation in a Ficoll-Hypaque gradient (PAA Laboratories, Pasching, Austria), as previously described [13]. After isolation, the cells were suspended in RPMI 1640 culture medium (BioWest, Nuaillé, France), (Ca^2+^)-free phosphate-buffered saline (PBS; Thermo Fisher Scientific, Waltham, MA, USA), or (Ca^2+^)-free Hanks’ balanced salt solution (HBSS; Sigma-Aldrich, St. Louis, MO, USA), depending on the test, and were stored at 4 °C until use. The buffy coats for the study were purchased from the Warsaw Regional Blood Centre. Peripheral venous blood for fractionation and preparation of buffy coats was collected in the centre from healthy male volunteers (ages 18–35 years). The donors were non-smokers, clinically recognised as healthy, and did not take any medications. The study complied with the principles of the Declaration of Helsinki. As it used a by-product material available on the market, it did not require the approval of the bioethics committee.

#### 3.4.2. Viability Assessment of Neutrophils and PBMCs

The potential cytotoxicity of the analytes was assessed by flow cytometry (BD FACSCalibur, BD Biosciences, San Jose, CA, USA) with propidium iodide (PI) staining and Triton X-100 solution as a positive control, according to Magiera et al. [13]. The final test concentrations were 1–100 µg/mL for the extracts/fractions and 5–50 µM for pure compounds. The analyses were performed after 24 h and 48 h of incubation for neutrophils and PBMCs, respectively.

#### 3.4.3. Evaluation of ROS, ELA-2, IL-8, IL-6, TNF-α and IL-10 Secretion by Human Immune Cells

All assays were performed according to the procedures described previously [13]. Briefly, the ROS production by neutrophils stimulated by *N*-formyl-l-methionyl-l-leucyl-l-phenylalanine (f-MLP) was determined using the luminol-amplified chemiluminescence assay. The ELA-2 secretion by *f*-MLP-cytochalasin B-stimulated neutrophils was determined spectrophotometric using *N*-succinyl-alanine- alanine-valine-p-nitroanilide (SAAVNA) as a substrate. The release of pro- and anti-inflammatory cytokines (IL-8, IL-6, TNF-α and IL-10) by the LPS-stimulated neutrophils and PBMCs was determined with enzyme-linked immunosorbent assays (ELISA) using the commercial kits. The secretion ranges of individual cytokines in ng/mL in stimulated and unstimulated controls for all donors are presented in Table 4. For each assay, appropriate sample dilutions were made to obtain the measuring ranges specified by the manufacturer. Finally, the secretion degree of a particular factor was calculated as a percentage compared to the control cell samples untreated by the tested analytes (100% activity). As positive controls, dexamethasone (5−50 µM) and quercetin (2.5−50 µM) were used. Before the experiments all extracts/compounds were dissolved in water–DMSO mixtures and diluted with the culture medium to obtain the desired concentrations. The final DMSO levels in the reaction environment were at most 2.5% and were checked not to influence the results. LPS (from Escherichia coli O111:B4) was purchased from Merck Millipore (Billerica, MA, USA), ELISA tests from BD Biosciences (San Jose, CA, USA), and all other reagents from Sigma-Aldrich (St. Louis, MO, USA). All measurements were performed in 96-well plates using a microplate reader (SYNERGY 4, BioTek, Winooski, VT, USA).

### 3.5. Statistics and Data Analysis

The results were expressed as means ± standard deviation (SD) for replicate determinations. The one-way analysis of variance (ANOVA), HSD Tukey test, and Dunnett’s test were applied to evaluate the statistical significance of the mean values, with *p* < 0.05 regarded as significant. The data were analysed using the Statistica13.1Pl software for Windows (StatSoft, Krakow, Poland).

## 4. Conclusions

The present study is the first comprehensive analysis addressing the phytochemical profile and biological capacity of dried *P. spinosa* fruits and their main components. Forty-five polyphenolic compounds and three MRPs, including ten new for the plant, were detected and structurally characterised using the LC-MS technique and NMR spectroscopy. Fractionated extraction enabled enrichment of the extracts in active constituents and activity enhancement. The obtained extracts/fractions significantly downregulated the pro-oxidant and pro-inflammatory functions of human neutrophils and PBMCs by modulating, in particular, the secretion of ROS, TNF-α, ELA-2, and IL-10. Polyphenols significantly contribute to the observed effects through the synergistic action of phenolic acids, condensed procyanidins, and flavonoids. The main constituent of the MRPs fraction, HMF, was nontoxic for the cells at a wide concentration range and able to significantly downregulate the TNF-α release from PBMCs acting thus additively with polyphenols. Compared to fresh sloes [13], dried fruits contain significantly lower levels of most polyphenolic compounds and are distinguished by the formation of MRPs. Nevertheless, dried sloes exhibit considerable pharmacological potential despite the decreased polyphenolic content. The present results might partly explain their traditional use as an inflammatory agent and indicate the extracts most suitable for functional application. However, further studies, including in vivo experiments, are required to confirm their effects eventually.

## Figures and Tables

**Table 1:** Quantitative profile of the *Prunus spinosa* dried fruit extracts (mg/g dw).

	MED	DEFD	EAFD	BFD	WRD
**Total contents:**					
TPC (GAE)	26.77 ± 0.47 ^d^	**124.01 ± 0.70 ^a^**	107.43 ± 4.08 ^b^	46.58 ± 2.28 ^c^	22.59 ± 0.05 ^d^
TPH	9.61 ± 0.60 ^d^	80.24 ± 1.16 ^b^	**109.91 ± 1.26 ^a^**	28.49 ± 0.96 ^c^	0.78 ± 0.01 ^e^
TPA	8.19 ± 0.51 ^d^	47.51 ± 1.50 ^b^	**102.53 ± 0.85 ^a^**	25.16 ± 0.80 ^c^	0.78 ± 0.01 ^e^
TAC	n.d.	n.d.	n.d.	**0.22 ± 0.01 ^a^**	n.d.
TFL	1.43 ± 0.09 ^d^	**25.99 ± 0.77 ^a^**	6.69 ± 0.46 ^b^	3.12 ± 0.24 ^c^	n.d.
TTC (PB2)	**8.17 ± 0.24 ^a^**	n.d.	n.d.	n.d.	5.37 ± 0.11 ^b^
MRPs	0.92 ± 0.07 ^c^	**37.75 ± 2.39 ^a^**	13.53 ± 0.78 ^b^	1.37 ± 0.03 ^c^	0.26 ± 0.003 ^c^
**Individual compounds:**					
Avicularin (**46**)	0.64 ± 0.09 ^b^	**4.44 ± 0.22 ^a^**	0.83 ± 0.14 ^b^	n.d.	n.d.
Guaiaverin (**45**)	n.d.	**0.91 ± 0.05 ^a^**	0.48 ± 0.03 ^b^	n.d.	n.d.
Hyperoside (**40**)	n.d.	n.d.	**0.84 ± 0.04 ^a^**	n.d.	n.d.
Isoquercitrin (**42**)	n.d.	0.13 ± 0.01 ^b^	**1.10 ± 0.14 ^a^**	n.d.	n.d.
Reinutrin (**44**)	n.d.	0.41 ± 0.003 ^b^	**0.71 ± 0.07 ^a^**	n.d.	n.d.
Rutin (**41**)	0.41 ± 0.03 ^c^	n.d.	0.74 ± 0.03 ^b^	**2.13 ± 0.20 ^a^**	n.d.
Quercitrin (**48**)	0.11 ± 0.01 ^c^	**1.40 ± 0.07 ^a^**	0.67 ± 0.03 ^b^	n.d.	n.d.
Quercetin (**53**, QU)	0.06 ± 0.01 ^c^	**16.41 ± 0.40 ^a^**	0.63 ± 0.04 ^b^	n.d.	n.d.
Cyanidin 3-*O*-glucoside (**24**)	n.d.	n.d.	n.d.	**0.22 ± 0.01 ^a^**	n.d.
Protocatechuic acid (**6**)	n.d.	**11.04 ± 0.96 ^a^**	0.57 ± 0.02 ^b^	n.d.	n.d.
*p*-Hydroxybenzoic acid (**11**)	n.d.	**1.47 ± 0.12 ^a^**	0.57 ± 0.04 ^b^	n.d.	n.d.
Vanillic acid (**19**)	n.d.	**7.85 ± 0.31 ^a^**	n.d.	n.d.	n.d.
*p*-Coumaric acid (**31**)	n.d.	**1.89 ± 0.18 ^a^**	n.d.	n.d.	n.d.
Neochlorogenic acid (**10**)	4.52 ± 0.34 ^d^	11.10 ± 0.40 ^c^	**29.28 ± 0.86 ^a^**	16.19 ± 0.50 ^b^	0.78 ± 0.01 ^e^
Chlorogenic acid (**20**, CHA)	0.14 ± 0.01 ^b^	0.28 ± 0.01 ^b^	**3.56 ± 0.21 ^a^**	0.20 ± 0.01 ^b^	n.d.
Cryptochlorogenic acid (**23**)	2.11 ± 0.03 ^c^	6.40 ± 0.40 ^b^	**50.85 ± 1.29 ^a^**	7.65 ± 0.49 ^b^	n.d.
Vanillin (**27**)	n.d.	**6.74 ± 0.47 ^a^**	0.69 ± 0.05 ^b^	n.d.	n.d.
5-Hydroxymethylfurfural (**2**, HMF)	0.78 ± 0.05 ^c^	**32.08 ± 2.03 ^a^**	11.50 ± 0.66 ^b^	1.16 ± 0.03 ^c^	0.22 ± 0.002 ^c^

Results are presented as means ± SD (*n* = 3). Numbers in parentheses (first column, in bold) refer to those in Figure 1 and Supplementary Table S1. For each parameter, different superscript letters (a–e) indicate significant differences (*p* < 0.05) in Tukey’s HSD test. MED, methanol-water (75:25, *v*/*v*) extract of dried fruits; DEFD, diethyl ether fraction of MED; EAFD, ethyl acetate fraction of MED; BFD, *n*-butanol fraction of MED; WRD, water residue of MED; TPC, total phenolic contents in gallic acid equivalents (GAE); TPH, total contents of low-molecular-weight phenols determined by HPLC-PDA; TPA, total phenolic acids; TAC, total anthocyanins; TFL, total flavonoids; TTC, total tannins in procyanidin B2 (PB2) equivalents. n.d.: below the quantitation (LOQ) or detection (LOD) limits.

**Table 2:** ^1^H NMR and ^13^C NMR spectral data of the compounds isolated from the fruits of *Prunus spinosa* (methanol-*d_4_*) ^a^.

Position/ Group ^b^	Compound 2		Compound 3	
	*δ*_C_	*δ*_H_	*δ*_C_	*δ*_H_
C-2	163.8	–	154.8	–
C-3	112.9	6.62 (1H, *d*, *J* = 3.1)	107.6	6.39 (1H, *d*, *J* = 3.0)
C-4	129.3	7.42 (1H, *d*, *J* = 3.1)	108.9	6.31 (1H, *d*, *J* = 3.0)
C-5	154.9	-	155.2	-
-OCHO-	-	-	98.2	5.41 (1H, *s*)
-CH_2_O-	58.2	4.62 (1H, *s*)	67.0	4.52 (2H, *s*)
-OCH_3_		-	55.9	3.37 (3H, *s*)
-CHO	182.6	9.56 (1H, *s*)	-	-

^a^ Data acquired with TMS as the internal standard, *δ* in ppm. Multiplicities and coupling constants (in Hz) are given in parentheses. ^b^ For trivial atom numbering, see Figure 3.

**Table 3:** Validation parameters for HPLC-PDA quantitative method.

Analyte	λ (nm)	Regression (Linear Model)		*r*	Linear Range (μg/mL)	*F*-Test	LOD (μg/mL)
		Equation	*n*				
HMF	285	y = 33981.70x − 15618.30	8	0.9999	1.08−108.0	167282.90	0.485

*λ*, detection wavelength; y, peak area; x, concentration of standard in (μg/mL); *n*, number of concentration levels (data points) used for construction of the regression equation; *F*-test, value of the statistical Fisher variance ratio for the experimental data, LOD, limits of detection.

**Table 4:** Ranges of cytokine secretion levels (ng/mL) in stimulated and unstimulated controls.

	Neutrophils		PBMCs		
	TNF-α	IL-8	TNF-α	IL-10	IL-6
stimulated control	0.5−1.8	43.9−88.8	1.5−5.8	1.4−2.8	2.3−2.7
unstimulated control	0.03−0.20	10.4−20.2	0.06−0.23	0.08−0.13	0.13−0.26

## Data Availability

Not applicable.

## Supplementary Materials

The following supporting information can be downloaded at: https://www.mdpi.com/article/10.3390/molecules27103302/s1. Table S1. UHPLC-PDA-ESI-MS*^3^* identification data of compounds detected in the dried fruit extracts of *P. spinosa*; Figure S1. Representative UHPLC chromatograms; Table S2. Quantitative profile of the *P. spinosa* fruit; Table S3. Correlation (*r*) coefficients and probability (*p*) values of linear relationships between antioxidant and anti-inflammatory activity parameters and phenolic contents of *P. spinosa* dried fruit extracts; Figure S2. Effect of the dried fruit extracts and pure compounds on viability (membrane integrity) of human immune cells. References [13,28,57,58,59,60,61,62,63,64,65,66,67,68] are cited in the supplementary materials.

## Sample Availability

Samples of the fruits and extracts are available from the authors.

## Abbreviations

BFD	*n*-butanol fraction of MED
BFF	*n*-butanol fraction of MEF
CHA	chlorogenic acid (5-*O*-caffeoylquinic acid)
CYG	cyanidin 3-*O*-*β*-d-glucopyranoside
DEFD	diethyl ether fraction of MED
DEFF	diethyl ether fraction of MEF
DEX	dexamethasone
dw	dry weight
EAFD	ethyl acetate fraction of MED
EAFF	ethyl acetate fraction of MEF
ELA-2	elastase 2 (neutrophils elastase)
*f*MLP	*N*-formyl-l-methionyl-l-leucyl-l-phenylalanine
fw	fresh weight
GAE	gallic acid equivalents
IL	interleukin
LOD	limits of detection
LOQ	limits of quantitation
LPS	bacterial lipopolysaccharide
MED	methanol-water (75:25, *v*/*v*) extract of dried fruits
MEF	methanol-water (75:25, *v*/*v*) extract of fresh fruits
MRPs	Maillard reaction products
MS^3^	three-stage mass spectrometry
PB2	procyanidin B2
PBMCs	peripheral blood mononuclear cells
QU	quercetin
ROS	reactive oxygen species
TAC	total content of anthocyanins
TFL	total content of flavonoids
TNF-α	tumour necrosis factor α
TPA	total content of phenolic acids
TPC	total phenolic content (Folin–Ciocalteu assay)
TPH	total phenolic content (sum of individual phenolics by HPLC)
TTC	total content of tannin-type proanthocyanidins
WRD	water residue of MED after fractionated extraction
WRF	water residue of MEF after fractionated extraction
