# Supplementary material for: Polyphenols and Maillard Reaction Products in Dried Prunus spinosa Fruits: Quality Aspects and Contribution to Anti-Inflammatory and Antioxidant Activity in Human Immune Cells Ex Vivo
Source: Molecules. 2022 May 20;27(10):3302. doi: 10.3390/molecules27103302 (PMC9143125; doi:10.3390/molecules27103302)
Supplement: Supplementary file 1 [file molecules-27-03302-s001.zip › molecules-1701740-supplementary.pdf]

molecules

Supplementary Material for:

Polyphenols and Maillard Reaction Products in Dried *Prunus spinosa* Fruits: Quality Aspects and Contribution to Anti-Inflammatory and Antioxidant Activity in Human Immune Cells *Ex Vivo*

Anna Magiera [1,*], Monika Ewa Czerwińska[2,3], Aleksandra Owczarek[1], Anna Marchelak[1], Sebastian Granica[4] and Monika Anna Olszewska[1]

[1] Department of Pharmacognosy, Faculty of Pharmacy, Medical University of Lodz, 1 Muszynskiego St., 90-151 Lodz, Poland
[2] Department of Biochemistry and Pharmacogenomics, Medical University of Warsaw, 1 Banacha St., 02-097 Warsaw, Poland
[3] Centre for Preclinical Research, Medical University of Warsaw, 1B Banacha St., 02-097 Warsaw, Poland
[4] Microbiota Lab, Centre for Preclinical Studies, Department of Pharmacognosy and Molecular Basis of Phytotherapy, Medical University of Warsaw, 1 Banacha St., 02-097 Warsaw, Poland
* Correspondence: anna.magiera@umed.lodz.pl (A. Magiera); Tel.: +48 503316997

Supplementary Material

Table of Contents

S.1. Results

Table S1. UHPLC-PDA-ESI-MS^3 identification data of compounds detected in the dried fruit extracts of *P. spinosa*.

Figure S1. Representative UHPLC chromatograms.

Table S2. Quantitative profile of the *P. spinosa* fruit.

Figure S2. Effect of the fruit extracts and pure compounds on viability (membrane integrity) of human immune cells.

Table S3. Correlation (*r*) coefficients and probability (*p*) values of linear relationships between antioxidant and anti-inflammatory activity parameters and phenolic contents of *P. spinosa* fruits extracts.

References

S.1. Results

Table S1. UHPLC-PDA-ESI-MS^3 identification data of compounds detected in the dried fruit extracts of *Prunus spinosa*.

Peak	Analyte	*Rt* (min)	UV λ_{max} (nm)	$[M–H]^-$ (m/z)	Fragmentary ions	$[M+H]^+$ (m/z)	Fragmentary ions	Extracts	References
1	3-Hydroxy-2,3-dihydromaltol (2,3-dihydro-3,5-dihydroxy-6-methyl-4(H)-pyran-4-one)	2.5	295	-	-	145	-	BFD, DEFD, EAFD, MED	[1]
2	5-Hydroxymethylfurfural (HMF)[a,b]	3.2	285	-	-	127	109	BFD, DEFD, EAFD, MED, WRD	[2, 3]
3	5-Hydroxymethoxymethyl-2-furanmethanol[b]	3.2	285	-	-	-	-	BFD, DEFD, EAFD, MED, WRD	-
4	Vanillic acid *O*-hexoside	3.9	250, 290	329	167	-	-	BFD, EAFD, MED, WRD	-
5	Protocatechuic acid 4-*O*-hexoside	4.3	280	315	153	-	-	EAFD	[4]
6	Protocatechuic acid[a]	4.6	260 295	153	153	155	155	DEFD, EAFD	-
7	Unidentified	5.2	325	517	335	-	-	BFD, MED, WRD	-
8	cis-3-*O*-Caffeoylquinic acid	5.7	322	353	191, 179	-	-	BFD, MED	[5]
9	3-*O*-Caffeoylquinic acid hexoside	6.2	325 295	515	353 (191, 179), 335, 341, 179			BFD, MED	[6]
10	3-*O*-Caffeoylquinic acid (neochlorogenic acid)[a]	6.7	325 265,211	353	191, 179, 135	-	-	BFD, DEFD, EAFD, MED, WRD	[7]
11	*p*-Hydroxybenzoic acid[a]	7.1	267 290	137	-	-	-	DEFD, EAFD	-
12	Unidentified	7.4	280	505	379	-	-	EAFD	-
13	(Epi)catechin derivative	7.5	280	415	289, 245	-	-	DEFD	-
14	Caffeoylshikimic acid derivative	8.0	-	505	335, 179	-	-	EAFD	[5]
15	Vanilloyl malate hexoside	8.9	260	445	329, 167	-	-	EAFD	-
16	3-*O*-*p*-Coumaroylquinic acid	9.2	310	337	163, 191	-	-	BFD, DEFD, EAFD, MED	[5]
17	*p*-Coumaric acid *O*-hexoside	9.7	280	325	163	-	-	EAFD	[8]
18	Amygdalin	10.1	210	456	323, 221	-	-	BFD	[9]
19	Vanillic acid[a]	10.1	216, 260, 290	167	167	169	169	DEFD	-
20	5-*O*-Caffeoylquinic acid (chlorogenic acid, CHA)[a]	10.6	325	353	191, 179	-	-	BFD, DEFD, EAFD, MED	[7]
21	*cis*-3-*O*-Feruloylquinic acid	11.0	215, 325, 293	367	193	-	-	DEFD, EAFD, MED	[5]
22	Unidentified	11.2	300, 225	415	293	-	-	BFD	-
23	4-*O*-Caffeoylquinic acid (cryptochlorogenic acid)[a]	11.5	325, 215	353	173, 191, 179	-	-	BFD, DEFD, EAFD, MED	[7]
24	Cyanidin 3-*O*-glucoside (CYG)[a]	11.6	515, 280	447	285, 401	449	287	BFD	-
25	Caffeic acid 3/4-*O*-hexoside	12.1	320	341	179, 135	-	-	DEFD, EAFD	-
26	3-*O*-Feruloylquinic acid	12.5	322	367	193	-	-	EAFD	[5]
27	Vanillin[a]	13.3	285	-	-	153	153	DEFD, EAFD	-
28	*cis*-3-*O*-*p*-Coumaroylquinic acid	14.6	305	337	163	-	-	DEFD, EAFD	[5]
29	Caffeoylquinic acid dehydrodimer	14.9	330, 287	705	513, 339	-	-	MED, WRD	[10]
30	4-*O*-Caffeoylshikimic acid	15.2	325	335	179, 135	-	-	DEFD, EAFD	[5]

31	*p*-Coumaric acid[a]	15.8	310	163	-	-	-	DEFD	-
32	4-*O*-Feruloylquinic acid	16.5	320, 217	367	176	-	-	EAFD	[5]
33	Caffeoylshikimic acid	17.4	280	335	161, 135, 179	-	-	BFD, DEFD, EAFD, MED	[5]
34	Caffeoylshikimic acid	18.9	215	335	161, 179	-	-	DEFD, EAFD	[5]
35	Unidentified	20.9	280	317	287	-	-	DEFD	-
36	*p*-Coumaroylshikimic acid	23.8	300, 205	319	119, 145, 275, 163, 257	-	-	DEFD, EAFD	[11]
37	Aromadendrin hexoside (dihydrokaempferol hexoside)	24.3	280, 328	449	287, 151, 269, 201	-	-	EAFD	[12]
38	Quercetin hexoside-pentoside	24.7	354	595	301, 433	-	303	BFD	-
39	*p*-Coumaroylshikimic acid	25.3	313, 290	319	145, 275, 257	-	-	DEFD, EAFD	[11]
40	Quercetin 3-*O*-*β*-D-galactoside (hyperoside)[a]	26.2	264, 355	463	301	-	303	EAFD	-
41	Quercetin 3-*O*-(6″-*O*-*α*-L-rhamnopyranosyl)-*β*-D-glucopyranoside (rutin)[a]	26.6	265, 350	609	301	-	303	BFD, EAFD, MED	-
42	Quercetin 3-*O*-*β*-D-glucopyranoside (isoquercitrin)[a]	27.7	260, 355	463	301	-	303	DEFD, EAFD	-
43	Quercetin 3-*O*-(2″-*O*-*β*-D-glucopyranosyl)-*α*-L-arabinofuranoside[a]	28.7	255, 355	595	433, 301	-	303	BFD, MED	-
44	Quercetin 3-*O*-*α*-D-xylopyranoside (reinutrin)[a]	30.1	256, 356	433	301	435	303	DEFD, EAFD	-
45	Quercetin 3-*O*-*α*-L-arabinopyranoside (guaiaverin)[a]	30.4	255, 355	433	301	435	303	DEFD, EAFD	-
46	Quercetin 3-*O*-*α*-L-arabinofuranoside (avicularin)[a]	35.1	255, 355	433	301	435	303	DEFD, EAFD, MED	-
47	Quercetin 3-*O*-(4″-*O*-*β*-D-glucopyranosyl)-*α*-L-rhamnopyranoside (multinoside A)[a]	35.8	254, 355	609	447, 301	611	449, 303	BFD, EAFD, MED	-
48	Quercetin 3-*O*-*α*-L-rhamnopyranoside (quercitrin)[a]	36.5	255, 355	447	301	449	303	DEFD, EAFD, MED	-
49	Quercetin malyl-pentoside	38.0	254, 350	549	433, 301	-	435, 303	EAFD	-
50	Quercetin malyl-pentoside	39.3	255, 355	549	433, 301	-	435, 303	DEFD	-
51	Kaempferol hexoside	42.0	255, 355	447	285	-	287	DEFD	-
52	Quercetin acetyl-hexoside-rhamoside	46.5	254, 350	651	609, 301	-	303	EAFD, MED	-
53	Quercetin (QU)[a]	49.2	255, 356	301	-	-	303	DEFD, EAFD, MED	-
54	Unidentified	50.9	350	337	322	-	-	DEFD	-

[a] Identified with authentic standards. [b] Identified by NMR. *Rt*, retention times. UV λ_{max}, absorbance maxima in UV-Vis spectra. $[M–H]^-$, deprotonated molecular ions in MS spectra recorded in a negative ion mode. $[M+H]^+$, protonated molecular ions in MS spectra recorded in a positive ion mode. Nomenclature of quinic acid esters including chlorogenic acid isomers is according to IUPAC rules adopted by Clifford et al. [7,13].

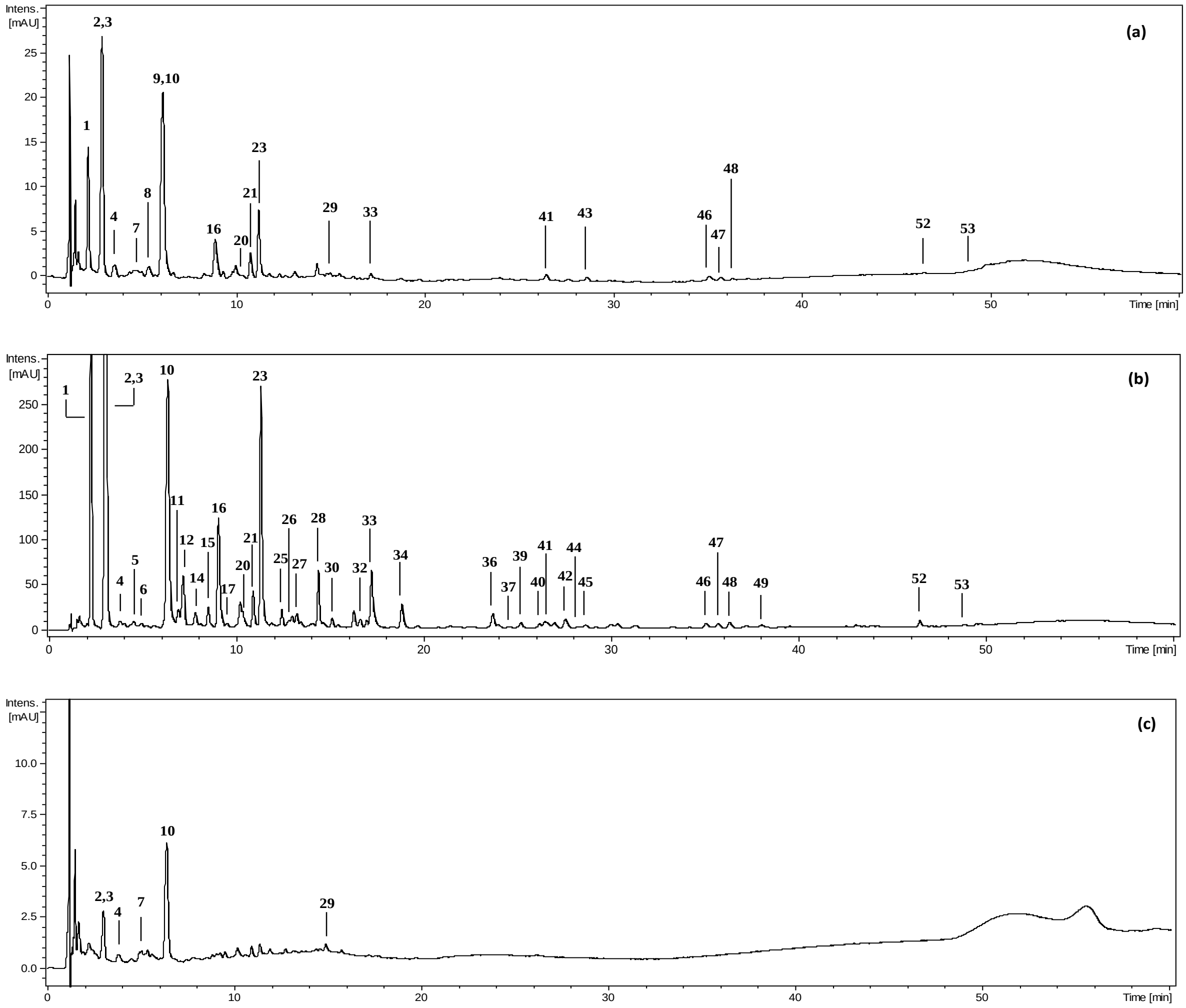

Figure S1. Representative UHPLC chromatograms at 280 nm of (a) methanol extract from dried fruits of *Prunus spinosa*, MED; ethyl acetate fraction from dried fruits, EAFD (b); and water residue from dried fruits, WRD. Peak numbers refer to those implemented in the Supplementary Table S1.

Table S2. Quantitative profile of the *Prunus spinosa* fruit.

	Dried fruit		Fresh fruit	
**Total contents**:	mg/g fw	mg/g dw	mg/g fw*	mg/g dw
TPC (GAE)	5.47 ± 0.09	13.43 ± 0.24	13.69 ± 0.55	33.63 ± 1.36
TPH	1.96 ± 0.12	9.61 ± 0.30	4.46 ± 0.09	10.97 ± 0.22
TPA	1.67 ± 0.10	4.11 ± 0.26	3.07 ± 0.05	7.55 ± 0.13
TAC	n.d.	n.d.	0.72 ± 0.02	1.78 ± 0.04
TFL	0.29 ± 0.02	0.72 ± 0.04	0.66 ± 0.03	1.63 ± 0.08
TTC (PB2)	1.67 ± 0.05	4.10 ± 0.12	6.96 ± 0.30	17.10 ± 0.74
MRPs	0.19 ± 0.01	0.46 ± 0.03	n.d.	n.d,
**Individual compounds**:				
Avicularin	0.13 ± 0.02	0.32 ± 0.04	0.21 ± 0.02	0.51 ± 0.05
Hyperoside	n.d.	n.d.	0.02 ± 0.0007	0.04 ± 0.001
Isoquercitrin	n.d.	n.d.	0.01 ± 0.0006	0.03 ± 0.001
Rutin	0.08 ± 0.006	0.20 ± 0.01	0.25 ± 0.003	0.61 ± 0.007
Quercitrin	0.02 ± 0.002	0.05 ± 0.005	0.02 ± 0.001	0.06 ± 0.004
Quercetin	0.01 ± 0.002	0.03 ± 0.005	n.d.	n.d.
Cyanidin 3-*O*-glucoside	n.d.	n.d.	0.31 ± 0.02	0.75 ± 0.05
Cyanidin 3-*O*-rutinoside	n.d.	n.d.	0.22 ± 0.01	0.53 ± 0.03
Peonidin-3-*O*-glucoside	n.d.	n.d.	0.15 ± 0.01	0.38 ± 0.01
Neochlorogenic acid	0.92 ± 0.07	2.27 ± 0.17	2.43 ± 0.05	5.96 ± 0.11
Chlorogenic acid	0.03 ± 0.002	0.07 ± 0.005	0.15 ± 0.003	0.36 ± 0.007
Cryptochlorogenic acid	0.43 ± 0.006	1.06 ± 0.01	0.24 ± 0. 01	0.60 ± 0.03
5-Hydroxymethylfurfural	0.16 ± 0.01	0.39 ± 0.03	n.d.	n.d.

Results are presented as means ± SD (n = 3). * Values according to Magiera et al. [14]. TPC, total phenolic contents in gallic acid equivalents (GAE); TPH, total contents of low-molecular-weight phenols determined by HPLC-PDA; TPA, total phenolic acids; TAC, total anthocyanins; TFL, total flavonoids; TTC, total tannins in procyanidin B2 (PB2) equivalents; fw, fresh weight; dw, dry weight. N.d.: below the limits of quantitation (LOQ) or detection (LOD).

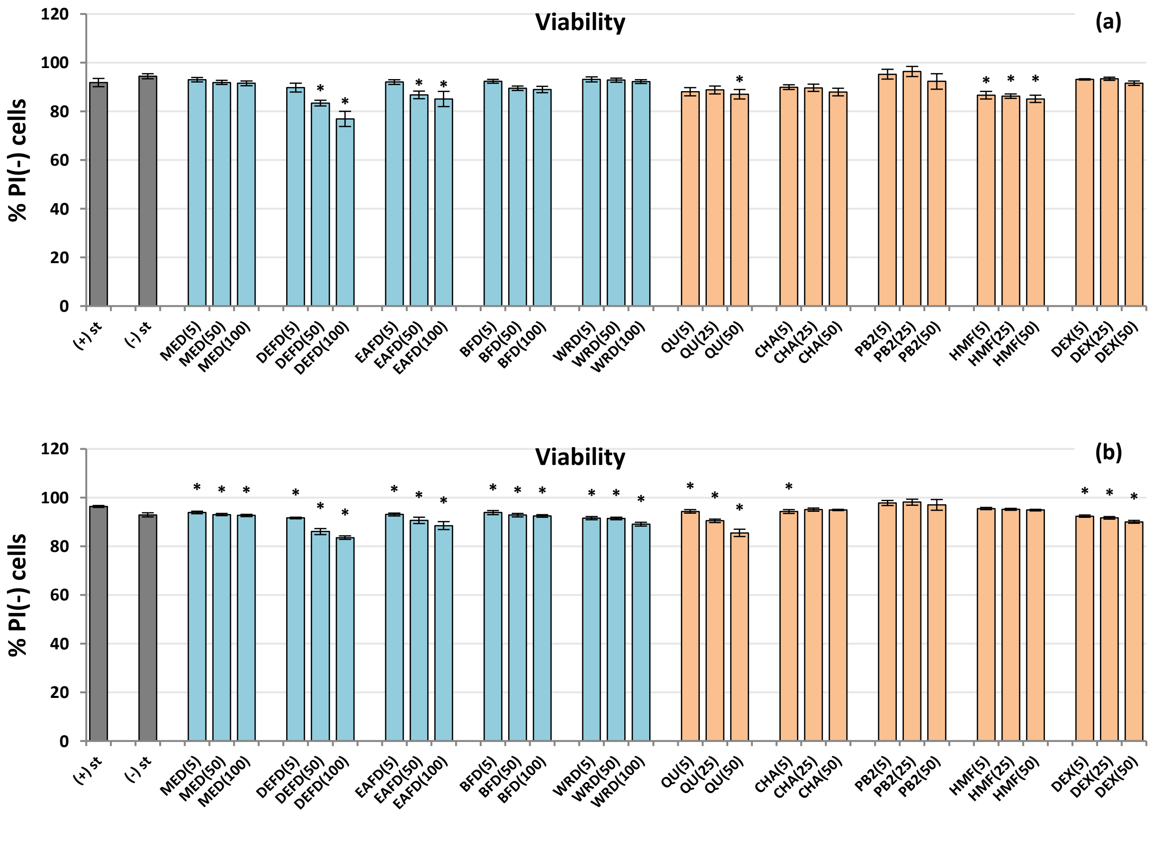

Figure S2. Effect of fruit extracts/fractions (5-100 μg/mL) and standards (5-50 μM) on viability (membrane integrity) of human immune cells expressed as a percentage of PI(-) cells (propidium iodide-negative): **(a)** effect on viability of neutrophils after 24 h incubation; **(b)** effect on viability of PBMCs after 48 h incubation. Extracts/fractions: MED, methanol-water (75:25, *v*/*v*) extract of dried fruits; DEFD, diethyl ether fraction of MED; EAFD, ethyl acetate fraction of MED; BFD, *n*-butanol fraction of MED; WRD, water residue of MED. Standards: DEX, dexamethasone; QU, quercetin; CHA, chlorogenic acid; PB2, procyanidin B2; HMF, 5-Hydroxymethylfurfural; Positive control: Triton X-100 solution (98.6% of PI(+) cells). Data expressed as means ± SD of five independent experiments performed with cells isolated from five independent donors. Statistical significance in Dunnett's test: *$p < 0.05$ compared with the stimulated control (+)st.

Table S3. Correlation (*r*) coefficients and probability (*p*) values of linear relationships between biological activity parameters and phenolic contents of *Prunus spinosa* fruits extracts.

		**Neutrophils**		
**r (*p*) for**	**ROS level**	**TNF-α secretion**	**ELA-2 secretion**	**IL-8 secretion**
TPC	-0.7099 (0.000)*	-0.5936 (0.020)*	-0.6390 (0.002)*	-0.1314 (0.641)
TPH+TTC	-0.6479 (0.001)*	-0.5292 (0.042)*	-0.6510 (0.001)*	-0.1317 (0.625)
		**PBMCs**		
**r (*p*) for**		**TNF-α secretion**	**IL-10 secretion**	**IL-6 secretion**
TPC		-0.9116 (0.000)*	0.5549 (0.032)*	-0.9378 (0.000)*
TPH+TTC		-0.9134 (0.000)*	0.6685 (0.006)*	-0.9774 (0.000)*

Values calculated using activity and concentration parameters reported in Table 1 and Figures 4-6. TPC, total phenolic contents in gallic acid equivalents (GAE); TPH, total contents of low-molecular-weight phenols determined by HPLC-PDA; TTC, total tannins in procyanidin (PB2) B2 equivalents. Asterisks mean statistical significance of the linear relationships ($p < 0$ 05).

References

1. Li, H.; Wu, C.-J.; Tang, X.-Y.; Yu, S.-J. Determination of four bitter compounds in caramel colors and beverages using modified QuEChERS coupled with liquid chromatography-diode array detector-mass spectrometry. *Food Anal. Methods* **2019**, *12*, 1674-1683.
2. Gökmen, V.; Senyuva, H.Z. Improved method for the determination of hydroxymethylfurfural in baby foods using liquid chromatography−mass spectrometry. *J. Agric. Food Chem.* **2006**, *54*, 2845-2849.
3. Teixidó, E.; Moyano, E.; Santos, F.J.; Galceran, M.T. Liquid chromatography multi-stage mass spectrometry for the analysis of 5-hydroxymethylfurfural in foods. *J. Chromatogr. A.* **2008**, *1185*, 102-108.
4. Catarino, M.D.; Silva, A.M.S.; Saraiva, S.C.; Sobral, A.B.J.F.N.; Cardoso, S.M. Characterization of phenolic constituents and evaluation of antioxidant properties of leaves and stems of *Eriocephalus africanus*. *Arab. J. Chem.* **2018**, *11*, 62-69.
5. Jaiswal, R.; Sovdat, T.; Vivan, F.; Kuhnert, N. Profiling and characterization by LC-MSn of the chlorogenic acids and hydroxycinnamoylshikimate esters in maté (*Ilex paraguariensis*). *J. Agric. Food Chem.* **2010**, *58*, 5471-5481.
6. Jaiswal, R.; Müller, H.; Müller, A.; Karar, M. G.; Kuhnert, N. Identification and characterization of chlorogenic acids, chlorogenic acid glycosides and flavonoids from *Lonicera henryi* L. (Caprifoliaceae) leaves by LC-MS^n. *Phytochemistry* **2014**, *108*, 252–263.
7. Clifford, M.N.; Johnston, K.L.; Knight, S.; Kuhnert, N. Hierarchical scheme for LC-MS^n identification of chlorogenic acids. *J. Agric. Food Chem.* **2003**, *51*, 2900-2911.
8. Bystrom, L. M.; Lewis, B.A.; Brown, D.L.; Rodriguez, E.; Obendorf, R.L. Characterization of phenolics by LC-UV/vis, LC-MS/MS and sugars by GC in *Melicoccus bijugatus* Jacq. 'Montgomery' fruits. *Food Chem.* **2008**, *111*, 1017–1024.
9. Xu, S.; Xu, X.; Yuan, S.; Liu, H.; Liu, M.; Zhang, Y.; Zhang, H.; Gao, Y.; Lin, R.; Li, X. Identification and analysis of amygdalin, neoamygdalin and amygdalin amide in different processed bitter almonds by HPLC-ESI-MS/MS and HPLC-DAD. *Molecules* **2017**, *22*, 1425.
10. Castillo-Fraireac, C.M.; Poupard, P.; Guilois-Dubois, S.; Salas, E.; Guyota, S. Preparative fractionation of 5′-O-caffeoylquinic acid oxidation products using centrifugal partition chromatography and their investigation by mass spectrometry. *J. Chromatogr. A.* **2019**, *1592*, 19-30.
11. Ben Said, R.; Hamed, A.I.; Mahalel, U.A.; Al-Ayed, A.S.; Kowalczyk, M.; Moldoch, J.; Oleszek, W.; Stochmal, A. Tentative characterization of polyphenolic compounds in the male flowers of *Phoenix dactylifera* by liquid chromatography coupled with mass spectrometry and DFT. *Int. J. Mol. Sci.* **2017**, *18*, E512.
12. Chen, G.; Li, X.; Saleri, F.; Guo, M. Analysis of flavonoids in *Rhamnus davurica* and its antiproliferative activities. *Molecules* **2016**, *21*, E1275.
13. Clifford, M.N.; Knight, S.; Kuhnert, N. Discriminating between the six isomers of dicaffeoylquinic acid by LC-MS^n. *J. Agric. Food Chem.* **2005**, *53*, 3821–3832.
14. Magiera, A.; Czerwińska, M.E.; Owczarek, A.; Marchelak, A.; Granica, S.; Olszewska, M.A. Polyphenol-enriched extracts of *Prunus spinosa* fruits: Anti-inflammatory and antioxidant effects in human immune cells ex vivo in relation to phytochemical profile. *Molecules* **2022**, *27*, 1691.
